# Regulation of early seedling establishment and root development in *Arabidopsis thaliana* by light and carbohydrates

**DOI:** 10.1007/s00425-023-04226-9

**Published:** 2023-09-06

**Authors:** Taras Pasternak, Stefan Kircher, Klaus Palme, José Manuel Pérez-Pérez

**Affiliations:** 1 Instituto de Bioingeniería, Universidad Miguel Hernández, 03202 Elche, Spain; 2 Faculty for Biology, Institute of Biology II/Molecular Plant Physiology, University of Freiburg, 79104 Freiburg, Germany; 3 Centre for BioSystems Analysis, BIOSS Centre for Biological Signalling Studies, University of Freiburg, 79104 Freiburg, Germany; 4 ScreenSYSGmbH, Engesserstr. 4a, Freiburg, 79108 Germany

**Keywords:** *Arabidopsis thaliana*, Carbohydrates, Cell differentiation, Cell division, Histone modifications, Root apical meristem

## Abstract

**Main conclusion:**

Root development is regulated by sucrose and light during early seedling establishment through changes in the auxin response and chromatin topology.

**Abstract:**

Light is a key environmental signal that regulates plant growth and development. The impact of light on development is primarily analyzed in the above-ground tissues, but little is known about the mechanisms by which light shapes the architecture of underground roots. Our study shows that carbohydrate starvation during skotomorphogenesis is accompanied by compaction of nuclei in the root apical meristem, which prevents cell cycle progression and leads to irreversible root differentiation in the absence of external carbohydrates, as evidenced by the lack of DNA replication and increased numbers of nuclei with specific chromatin characteristics. In these conditions, induction of photomorphogenesis was unable to restore seedling growth, as overall root growth was compromised. The addition of carbohydrates, either locally or systemically by transferring seedlings to sugar-containing medium, led to the induction of adventitious root formation with rapid recovery of seedling growth. Conversely, transferring in vitro carbohydrate-grown seedlings from light to dark transiently promoted cell elongation and significantly reduced root meristem size, but did not primarily affect cell cycle kinetics. We show that, in the presence of sucrose, dark incubation does not affect zonation in the root apical meristem but leads to shortening of the proliferative and transition zones. Sugar starvation led to a rapid increase in lysine demethylation of histone H3 at position K9, which preceded a rapid decline in cell cycle activity and activation of cell differentiation. In conclusion, carbohydrates are required for cell cycle activity, epigenetics reprogramming and for postmitotic cell elongation and auxin-regulated response in the root apical meristem.

**Supplementary Information:**

The online version contains supplementary material available at 10.1007/s00425-023-04226-9.

## Introduction

Under natural conditions, the seeds are normally buried underground, and the seedlings germinate using only their internal resources, i.e., hormones and carbohydrates. Proper seedling establishment requires cotyledon expansion and greening, as well as a metabolic shift toward auxotrophic development triggered by the reactivation of photosynthesis before the internal resources are exhausted. The mature seeds of *Arabidopsis thaliana* (hereinafter Arabidopsis) lack carbohydrates but contain high levels of fatty acids (Baud et al. 2008), whose hydrolysis through specific lipases provides intermediates for the synthesis of sugars through gluconeogenesis (Quettier and Eastmond 2009). During seedling growth in the dark, known as skotomorphogenesis, the limited internal resources of the seed are directed primarily toward elongation of the hypocotyl to push the cotyledons to the soil surface and allow autotrophy, while the development of the primary root (PR) is, therefore, limited (Franklin and Quail 2010). After the initiation of the light-dependent photomorphogenic program, PR growth is enhanced to ensure the uptake of water and nutrients necessary to sustain further seedling growth.

Most of the laboratory studies aimed at investigating the growth and development of Arabidopsis roots have generally been carried out in nearly vertically oriented plates grown in light and with the presence of carbohydrates (sucrose) in the culture medium. These non-natural conditions can bias the interpretation of the results thus obtained (Cabrera et al. 2022), among those due to the contribution of the sucrose signaling pathway to the regulation of root architecture (Mudgil et al. 2016). The effect of carbohydrates on root morphogenesis was proposed earlier in 1922 (Robbins 1922) and has been extensively investigated in Arabidopsis seedlings thereafter. Kircher and Schopfer (2012) have shown that photosynthetic carbohydrates act as long-distance signaling molecules to drive root growth and development in young seedlings. To the best of our knowledge, important aspects of PR growth, such as the relationship between cell cycle progression, cell expansion, chromatin organization and the role of exogenous carbohydrate addition, have yet to be investigated in detail under different light and dark conditions. Furthermore, the epigenetic aspects of light/dark regime and the effect of carbohydrates on the balance between cell division and cell elongation are fundamental for root growth and development. Recent investigations based on the analysis of histone modifications provide additional evidence on this regulation in both the animal and plant kingdoms (Morao et al. 2016; Pasternak et al. 2020).

Auxin is a key hormone for plant growth and development and acts as a functional integrator of endogenous and exogenous signals in the root developmental pathway (Lavenus et al. 2013; Liu and von Wirén 2022). Arabidopsis seeds have a large pool of auxin in the form of amide-linked IAA conjugates, which are actively deconjugated by hydrolases during germination (Rampey et al. 2004). However, during early seedling growth, endogenous auxin is directly dependent on photosynthesis-derived IAA which is synthesized in the aerial organs (young leaves and cotyledons) and is transported polarly through the hypocotyl and into the root via the aid of the PIN-FORMED (PIN) auxin efflux facilitators (Mano and Nemoto 2012; Adamowski and Friml 2015). Thus, carbon starvation during skotomorphogenesis may indirectly prevent the establishment of de novo auxin biosynthesis in a tissue-specific manner. Recent data have shown that sucrose acts as a light-induced mobile signal from shoots to synchronize lateral root (LR) formation, in part by promoting local auxin biosynthesis at the root tip (Kircher and Schopfer 2023).

By detailed analysis of polar auxin transport and auxin responses, analysis of cell cycle kinetics, of cell and nucleus geometry, and of euchromatin and heterochromatin quantification, we demonstrate that carbohydrates are the major limiting factors for the effective establishment of Arabidopsis seedlings after germination. Our results indicate that lack of carbohydrates is a key negative factor during Arabidopsis seedling development, and that proper adventitious root (AR) formation from the hypocotyl allows recovery of seedling viability, which is dependent on the presence of photosynthesis-derived or exogenously applied carbohydrates in this species.

## Materials and methods

### Plant material, growth conditions and treatment

The plant genotypes used in these experiments are wild type *Arabidopsis thaliana* (L.) Heyhn. Columbia-0, *suc2* (Gottwald et al. 2000), and *phyABcry12* mutants (Yanovsky et al. 2000), CyclinB1;1-GUS (Colón-Carmona et al. 1999), DII-VENUS (Brunoud et al. 2012); DR5::GFP; TAA1::GFP and YUCCA1::GUS lines have been described elsewhere (Pasternak et al. 2022). The seeds were surface-sterilized and distributed in square Petri dishes with TK1 medium (Pasternak et al. 2023) supplemented with 150 mg/L Bacto Tryptone (Roth, Karlsruhe, Germany), 0 or 1% sucrose, 5 mM MES and 1.1% agar (Roth) and set at a pH 5.6. Seeds sown on these plates were kept at room temperature for 4 h to allow water uptake. After sowing, the seeds were soaked at 4 °C for 12 h and exposed to white light (100 µmol m^−2^ s^−1^) for 8 h before being transferred to final growth conditions. The seedlings were grown in plates placed vertically in an incubator maintained at 24 °C in continuous white light with an intensity of 100 µmol m^−2^ s^−1^. For dark growth conditions, the plates were covered with black tissue and placed in a black box in the same growth chamber. The days of growth were counted after transferring the 4 °C Petri dishes to the growth chamber. Petri dishes were periodically scanned with an Adobe 950 Scanner and root length was measured with Image J software (Schneider et al. 2012). Glucose and Fructose were prepared as 10% stock, filter.sterlized and added to to agar-conatined medium after autoclaving. The different conditions and treatments used are indicated in Suppl. Fig. S1.

### Quantification of fluorescence signal and statistics

Images from the seedlings growing on vertical plates have been taken with Zeiss AxioCamMRc camera under a ZEISS SV11 APO stereomicroscope equipped with fluorescent HBO lamp with GFP filter set (488 nm excitation and 530–550 nm emission) and further processed with Axiovision Rel 4.8 imaging software. Total magnification of 250 × has been used. To compare the data for a given variable, we performed multiple testing analyses with the ANOVA *F*-test or the Fisher’s Least Significant Difference (LSD) post hoc test, as indicated. Significant differences were defined as a 1% level of significance (*P*-value < 0.01) unless otherwise indicated. Experiments were repeated twice, and quantitative measurements were done by measuring mean gray levels of the green channel of each image using ImageJ software.

### Whole mount in situ immunolocalization

Immunolocalization in Arabidopsis seedlings was performed according to a whole mount in situ protocol (Pasternak et al. 2015). Affinity-purified primary anti-PIN1 (10A7, mouse), anti-PIN2 (P192, Guinea pig); anti-PIN4 (5404, rabbit), anti-histone H3K9me2 (di methyl Lys9) antibody (mAbcam 1220, anti-mouse), anti-histone H3K4me2 (di-methyl Lys4) antibody (GenTex Cat. No. GTX121915, anti-rabbit) antibodies were diluted 1:40 (PIN1) and 1:200 for all others. Alexa-488/Alexa-555-conjugated anti-mouse or anti-rabbit secondary antibodies were diluted 1:400.

### Histochemical GUS assay

The activities of the *DR5::GUS* and *CyCB1;1::GUS* reporter were determined by a standard GUS histochemical staining procedure. Seedling were fixed in cold acetone for 5 min and then placed in the GUS staining solution: 1 mg mL^−1^ X-Gluc in 50 mM NaP buffer (pH 7.0), containing 1 mM EDTA and 2 mM ferric cyanide. Incubation for 4–12 h was performed at 37 °C and the explants were immersed in methanol thereafter. After 15 min of incubation, distilled H_2_O (dH_2_O) was gradually added until a final methanol concentration of 15% was obtained. The samples were washed twice with dH_2_O and subsequently rinsed in 8:3:1 chloral hydrate: dH_2_O: glycerol (by vol.) for 10 min. Finally, the seedlings were mounted in 50% glycerol on glass microscope slides. Samples were visualized using Nomarski optics on an Axioplan 2 microscope (Zeiss, Oberkochen, Germany), using Axiovision 4.8 software (Zeiss, Jena, Germany) for image processing.

### Cell boundary and starch stain

For 3D root analyses, a method allowing staining of cell borders and simultaneous sensitive detection of starch grains was employed by modifying the procedure described by Truernit et al. (2008) as follows. Seedlings were fixed as described above for immunolocalization, washed with dH_2_O, treated 25 min in methanol, and finally slowly rehydrated in dH_2_O. Seedlings were then incubated for 20 min in 1% periodic acid, washed twice with dH_2_O, and stained with 4 mg/L propidium iodine solution in 100 mM Na_2_SO_3_ (pH 1.4 adjusted with HCl, final concentration of about 0.15 M). The seedlings were mounted in chloral hydrate solution for microscopy analyses on slides with a 100 µm spacer for the preservation of their 3D structure.

### Analysis of cell cycle kinetics

Cell cycle processes were analyzed according to Pasternak et al. (2022).

### Confocal microscopy

Images were acquired using a Zeiss LSM 510 NLO and Leica 5 confocal scanning microscopes. The excitation wavelengths were 488 nm (argon laser) for Alexa 488-conjugated antibodies and GFP, and 543 nm (Helium–Neon-Laser) for Alexa 555-conjugated antibodies and propidium iodine. Emission was detected at 500–550 nm for A488-conjugated antibodies and GFP, and above 575 nm for A555-conjugated antibodies and propidium iodine. DAPI (4',6-diamidino-2-phenylindole) images were obtained using a 2-photon module with excitation at 2 × 365 nm and emission at 435–485 nm. All multi-labelled signals were detected in multi-tracking mode to avoid fluorescence crosstalk. Images were analyzed with the ZEN2010 image browser (Carl Zeiss Micro Imaging), and LEICA LAZ ES and Image J.

### Nucleus positions and volume determination

To determine the positions of the nuclei and their volumes, the roots after fixation were washed with dH_2_O, treated with methanol, rehydrated in dH_2_O, and rinsed in chloral-hydrate. The cleared samples were labeled with DAPI and scanned using a 2-photon confocal microscope with an excitation wavelength of 730 nm and an emission band of 435–485 nm using a × 40 oil immersion objective. The recorded tiles were stitched together using the XUV stitcher tool (Emmenlauer et al. 2009). The position of each nucleus in the root was determined automatically and the nucleolus volumes in the trichoblast cell files were manually annotated using the iRoCS labelling tool (Pasternak and Pérez-Pérez 2021). The structures of the nuclei were analyzed according to Dubos et al. (2020, 2022).

## Results

### Sucrose is required for PR growth during early seedling establishment

**Fig. 1:**
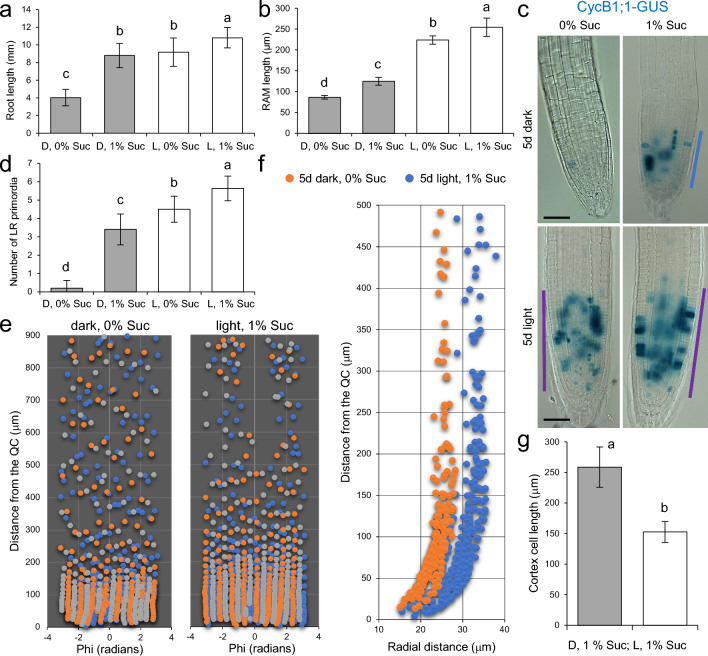
Sugar is required for PR growth during early seedling establishment. **a**, **b** Root and RAM length were measured in seedlings grown for 5 days in the medium with and without 1% sucrose, under 0 (Dark, D) or 100 µmol m^−2^ s^−1^ continuous light (Light, L). **c** Expression of the CycB1;1-GUS marker (shown in blue) in the same conditions as indicated above. The purple line indicates the region of the PZ of the RAM. **d** Number of LR primordia in the same conditions as indicated above. **e** Quantitative analysis of the RAM structure under dark and light conditions without sucrose. Seedlings were grown 3 days under dark or light conditions, fixed, segmented and a cellular map of the outer cell layers was obtained as described elsewhere (Pasternak and Pérez-Pérez 2021). Orange: cortex cells, blue: atrichoblasts, gray: trichoblasts. **f** Quantitative analysis of the cortex layer in a representative sample across spatial coordinates. **g** Length of cortex cells in the mature zone. The bars in **a**, **b**, **d** and **g** show the mean values ± standard deviation (SD), *n* = 10. Letters indicate significant differences between treatments (*P*-value < 0.01; Least Significant Difference [LSD]). Scale bars = 40 μm

Presence of 1% sucrose in the culture medium in 5-day-old seedlings grown in the dark restored PR length to levels like those in the light-grown seedlings without sucrose (Suppl. Fig S1 and Fig. 1a). The addition of sucrose to dark-grown seedlings produced a slight extension of the root apical meristem (RAM) to a lesser degree than that observed in seedlings grown in light without sucrose (Fig. 1b). In agreement with this, we found that CycB1;1-GUS fusion protein, used here as a cell division marker (Colón-Carmona et al. 1999), did not accumulate in the proliferation zone (PZ) of the RAM in seedlings grown in the dark without sucrose (Fig. 1c). However, in light-grown seedlings, CycB1;1-GUS accumulated over a broader region within the PZ of the RAM than in dark-grown seedlings, and at similar levels with and without sucrose in light-grown seedlings (Fig. 1c). Interestingly, in seedlings grown in the dark in the presence of sucrose, CycB1;1-GUS was detected in the RAM but in a smaller region compared to seedlings that had been grown in light conditions (Fig. 1c, blue lines). In addition, sucrose significantly increased the number of LR primordia either in dark or in light conditions (Fig. 1d).

To investigate the mechanism of sucrose-mediated PR-restorative growth in seedlings grown in the dark, we analyzed the organization of the RAM using the Deep-Resolution Plant Phenotyping Platform pipeline (Schmidt et al. 2014; Pasternak and Pérez-Pérez 2021) at an intermediate point during development (3 days). Virtual cross sections of the PRs confirmed that the radial structure of the RAM is conserved in dark- and light-grown seedlings (Fig. 1e), although roots were substantially thinner when grown in dark conditions without sucrose (Fig. 1f). At the cellular level, the main difference between both conditions is the reduction in the size of the PZ due to a low ratio of replicating and mitotic cells in the PZ of dark-grown seedlings (Suppl. Fig. S2a, b). As an additional marker of premature differentiation within the RAM in dark-grown seedlings, we observed starch granules in the inner tissues of the cortex and endodermis in a distal region and near the root tip (Suppl. Fig. S2c), which correlated with the significant increase in the size of the cortex cells in the elongation zone (EZ) of the RAM (Fig. 1g).

**Fig. 2:**
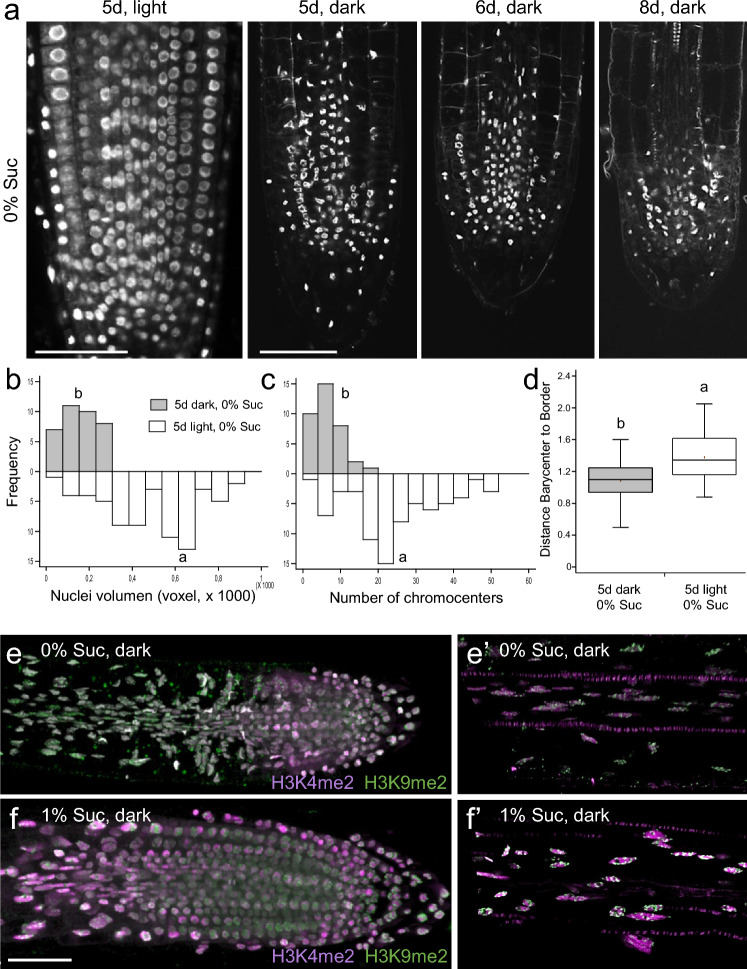
Sugar is required to maintain functional chromatin in the RAM. **a** Details of nuclei structure in the proximal RAM as stained by DAPI after 5, 6 and 8 days in light or in dark conditions without 1% sucrose. **b–d** Quantitative analysis of nuclei parameters. Volume (**b**), number of chromocenters (**c**) and mean distance from barycenter to border (**d**) were estimated as using the NucleusJ 2.0 plugging (Dubos et al. 2020); *n* = 600. **e**, **f** Chromatin features in the RAM (**e**, **f**) and the differentiation zone (**e′**, **f′**) in response to sugar. Seedlings were cultured in the dark in the absence (**e**, **e’**) and presence (**f**, **f’**) of 1% sucrose. DAPI staining is shown in white, H3K4me2 in purple and H3K9me2 in green. Letters in **b–d** indicate significant differences between treatments (*P*-value < 0.01; LSD). Scale bars = 50 μm

Extending the length of the dark incubation period without sucrose to 8 days produced a concomitant reduction of the proliferative region of the RAM, which was associated with visual alteration of the characteristics of the nuclei in these cells (Fig. 2a). Cell nuclei within the RAM in 5-day-old seedlings grown under light or dark conditions were segmented and various parameters including volume, elongation and heterogeneity were estimated. DAPI staining allowed us to separate the nucleolus compartment based on its low affinity for RNA. Cell nuclei within the RAM of dark-grown seedlings without sucrose were characterized by their smaller volumes, reduced nucleoli size, and lower values of nuclear heterogeneity compared to those of light-grown seedlings (Fig. 2b–d). We wondered if the changes in the nucleus morphology that we have observed were associated with histone modifications at the chromatin level. We chose two histone methylation marks, H3K4me2 and H3K9me2, which have been previously associated with some regions of euchromatin (Zhang et al. 2009) and heterochromatin (Zhang et al. 2023), respectively. H3K4me2 was found at higher levels in the PZ of the RAM in seedlings grown in the dark with sucrose, but lower levels of H3K4me2 were observed in seedlings grown without sucrose in this region (Fig. 2e, f). In both cases, H3K4me2 was mostly absent in the differentiation zone of the root and was found in a more distal region in dark-growing seedlings without sucrose (Fig. 2e). Consistent with the known role of H3K9me2 in the formation of constitutive heterochromatin, speckles of H3K9me2 immunolocalization were found in almost all cells within root tips, even in the EZ (Fig. 2e′, f′). Interestingly, the addition of sucrose was able to restore proliferative characteristics associated with cell division in the distal RAM (Fig. 2f).

These results suggest that dark-induced carbon starvation reduced the proliferative potential of cells within the RAM and promotes their premature differentiation soon after gemination, a phenotype that can be reversed by adding sucrose to the growth medium.

### Effect of the sucrose transport and photomorphogenesis on PR growth

We have shown that PR growth of the *suc2* sucrose transport mutant (Gottwald et al. 2000) and the *phyA phyB cry1 cry2* quadruple photoreceptor mutant (Yanovsky et al. 2000) was reduced under light conditions in the absence of sucrose compared with the WT background, indicating that photosynthesis in the apical region of the seedling and sucrose transport through the phloem to the PR are required to sustain root growth (Suppl. Fig. S3).

### Light and sucrose are required to establish endogenous auxin levels in the RAM

**Fig. 3:**
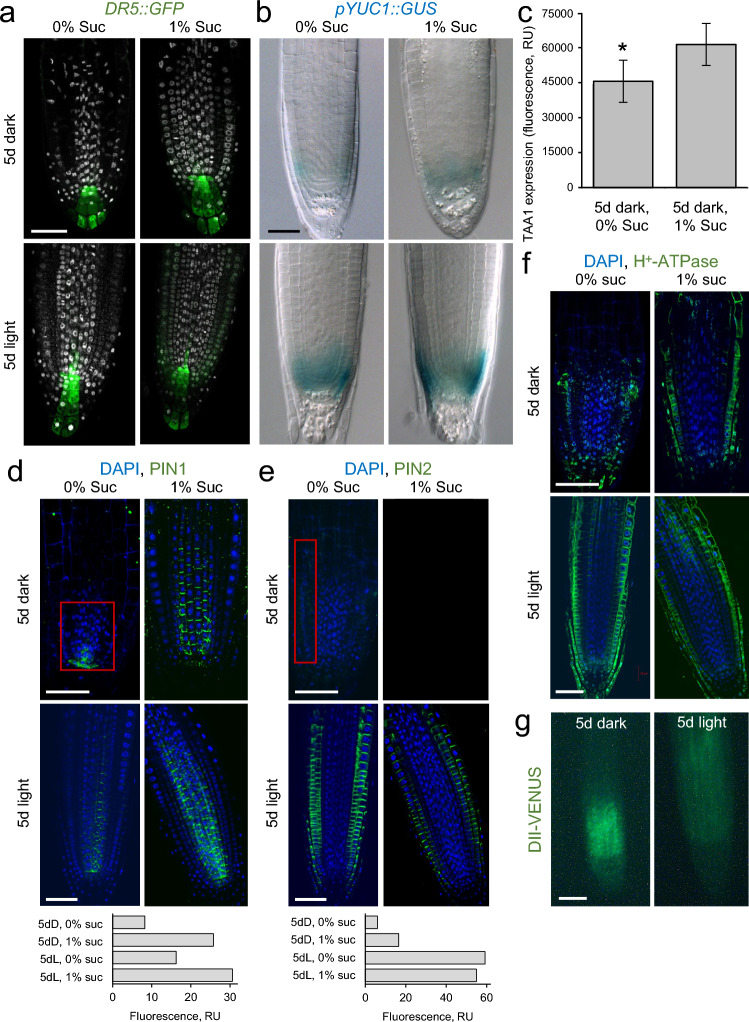
**a–c** Effect of light and sucrose on auxin homeostasis in the RAM during early seedling establishment. *DR5::GFP* (green, **a**), expression of the auxin biosynthesis genes (**b**, *pYUC::GUS*, blue), and overall *TAA1* expression levels (**c**) in the RAM estimated from fluorescence. RU, relative units. **d–f** Abundance of the PIN1 (**d**) and PIN2 (**e**) auxin efflux facilitators shown in green. H^+^-ATPase in **f** as a RAM integrity marker. DAPI staining of nuclei is shown in blue. To quantify PIN1 and PIN2, GFP fluorescence was measured in relative units (RU) in a given area (shown in red). **g** Overall *DII-VENUS* expression in the RAM shown in green. Seedlings have been grown for 5 days under dark or light conditions in the presence or absence of 1% sucrose, as indicated. Scale bars = 40 μm

Auxin is a key regulator of root growth and development. In the RAM, the proliferative potential of the tissue initials is maintained by local auxin biosynthesis and polar auxin transport, which together contribute to the establishment of an endogenous auxin gradient, with a maximum in the quiescent center (QC) region (Brumos et al. 2018). The auxin response marker *DR5::GFP* was expressed at similar levels in the root tip of seedlings grown for 5 days under dark or light conditions and independently whether sucrose was present in the medium (Fig. 3a). However, the activity of the *YUCCA1* promoter (*pYUC1::GUS*) in the root tip was clearly increased in the light-growing seedlings, but hardly increased in the root tip of the seedlings grown in the dark with sucrose (Fig. 3b). Conversely, *pTAA1::GFP* expression (Stepanova et al. 2008) was slightly increased in the root tip of seedlings grown in the dark with sucrose (Fig. 3c).

We indirectly monitored the intensity of polar auxin transport by immunolocalizing PIN1 and PIN2 auxin efflux facilitators, which are key to establishing the “reflux loop” of auxin required for PR growth and gravitropism (Blilou et al. 2005). We observed a lower abundance of PIN1 and PIN2 proteins in the RAM of seedlings grown in the dark without sucrose, and a positive effect of sucrose to increase PIN1 and PIN2 protein levels in the dark and PIN1 levels also in the light (Fig. 3d, e). Interestingly, the plasma membrane H^+^ ATPase used here as a reference for RAM integrity was detected at lower levels in seedlings grown in the dark without sucrose (Fig. 3f). Taken together, these results suggest that the establishment of an endogenous auxin gradient within the RAM is compromised in seedlings grown in the dark without sucrose due to a reduction in both local auxin biosynthesis and polar auxin transport from the shoot. Interestingly, we found that the agravitropic phenotype of *pin2* mutants grown in the light was restored by incubating seedlings in darkness with 1% sucrose (Suppl. Fig. S4). In fact, higher expression of the DII-VENUS sensor of auxin distribution (Brunoud et al. 2012) was found in the RAM of dark-grown seedlings without sucrose, indicating their lower levels of endogenous auxin response (Fig. 3g).

### Endogenous sugar levels are required for sustained cell proliferation and cell growth in the RAM and for seedling establishment

**Fig. 4:**
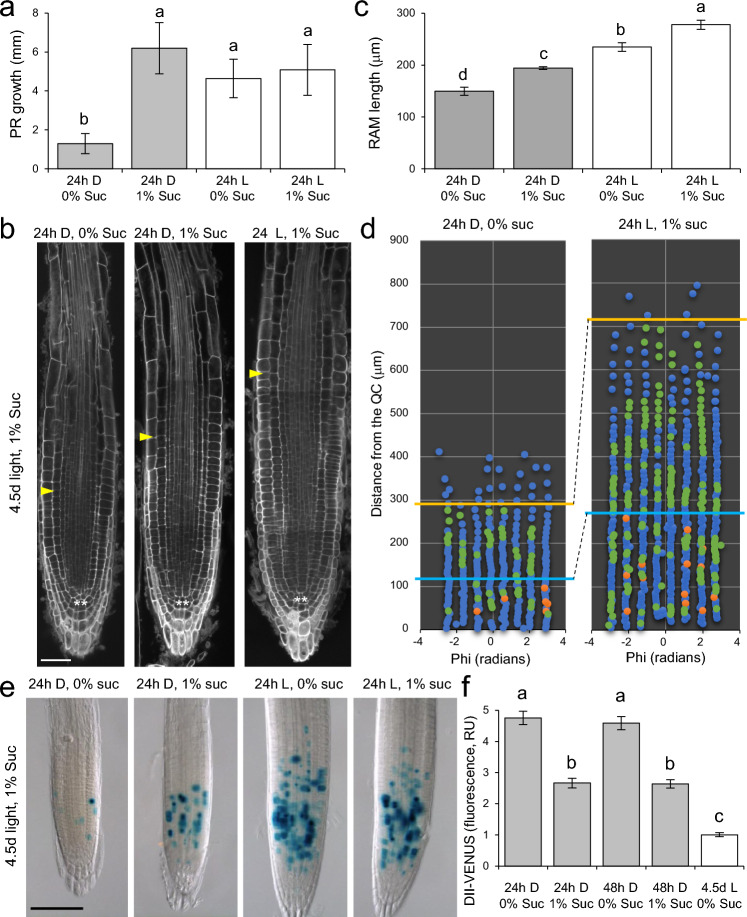
Post-germinative carbon starvation affects PR growth. **a–c** PR growth (**a**, **b**) and RAM length (**c**) in seedlings grown on 1% sucrose medium for 4.5 d in the light and that have been transferred to liquid medium under dark (D) or light (L) conditions with 0% or 1% sucrose. Arrow in **b** indicates the end of the PZ of the RAM estimated by the increased volume of cortical cells; asterisks mark the QC cells. **d** DNA replication and mitosis events of cortical cells in a representative root sample. 4.5-day-old seedlings were cultured for 24 h in the dark/light conditions, EdU/colchicine were added for the last 90 min. EdU (green) and mitotic figures (orange) were detected and analyzed as previously described (Pasternak et al. 2022). Blue lines and orange lines indicate the limit of PZ and EZ, respectively. **e** Expression of the CycB1;1-GUS marker (blue) in the same conditions as indicated in (**a**). **f** Overall *DII-VENUS* expression levels in the RAM estimated from fluorescence. RU, relative units. The bars in **a**, **c** and **f** show the mean values ± SD (*n* = 10). Letters indicate significant differences between treatments (*P*-value < 0.01; LSD). Scale bars = 40 μm

To investigate the effect of carbon starvation on root growth and seedling development, we transferred seedlings grown for 4.5 days in light with 1% sucrose to liquid medium under dark or light conditions with and without sucrose (Suppl. Fig. S1). Further PR growth was severely inhibited after 24 h of dark incubation without sucrose (Fig. 4a), resulting in smaller RAM sizes (Fig. 4b, c), due to a reduction in the number of cells that they replicate DNA, (Fig. 4d and Suppl. Fig. S5a). No significant PR growth was observed in the absence of sucrose in the medium, regardless of the incubation time in the dark (Suppl. Fig. S5a). On the other hand, the duration of incubation in the dark increased the growth of the hypocotyl in a sucrose-dependent manner, which is primarily dependent on cell expansion (Suppl. Fig. S5b). In agreement with these results, the number of cells that express the cell division marker CycB1;1-GUS was severely reduced soon after carbon starvation, an effect that can be partially compensated by the addition of sucrose, but to lower levels than those of seedlings incubated for 24 h in the light, regardless of the addition of sucrose (Fig. 4e). Furthermore, we found that carbon starvation also reduced PIN1, PIN2 and PIN7 protein levels, but not of PIN4, in the RAM, in a sucrose-dependent manner (Suppl. Fig. S5c, d). Endogenous auxin levels were monitored by DII-VENUS expression (Fig. 4f). DII-VENUS expression was significantly reduced in the RAM of seedlings incubated in the dark with sucrose for a short period as regards those in dark-grown seedlings without sucrose (Fig. 4f), indicative of an increase in auxin levels by sucrose addition in dark-grown seedlings. These results suggest that sucrose is continuously required to maintain auxin homeostasis in the PR and for sustained cell proliferation and endoreduplicative cell growth in the RAM.

**Fig. 5:**
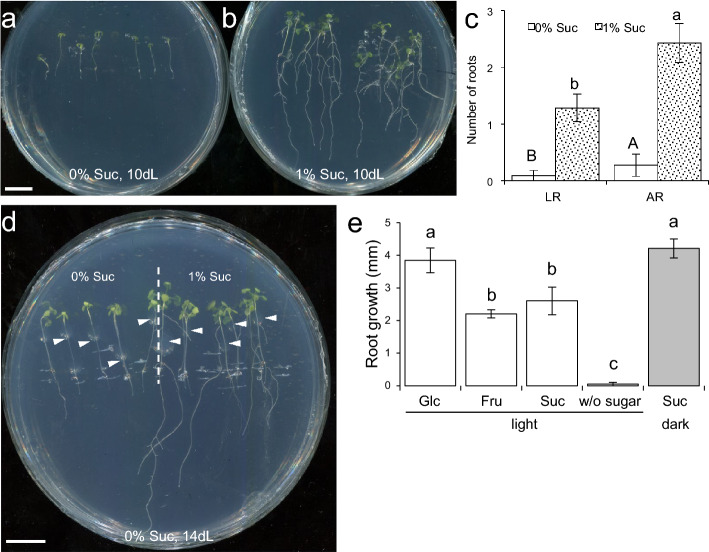
Sugar-induced recovery of seedling growth after carbon starvation. **a**, **b** Seedlings grown for 4.5 days in the dark without sucrose were transferred to plates supplemented with 0% (**a**) or 1% (**b**) sucrose. Images of whole seedlings were obtained after 10 days of growth in continuous light (10dL). **c** Number of ARs and LRs of seedlings in (**a**, **b**) at 10 dL. **d** Local application of sucrose (white arrowheads) after 4.5 days of growth in the dark without sucrose. Images in **d** were scanned after 14 days under continuous light (14 dL). **e** Sugar-mediated root growth recovery after carbon starvation. Seedlings were grown on the surface of agar medium without carbon and thereafter were transferred to the medium containing 1% of filter-sterilized glucose (Glc), fructose (Fru), or sucrose (Suc). New root growth has been measured after 90 h. The bars in **c**, **e** show the mean values ± SD (*n* = 10). Letters indicate significant differences between treatments (*P*-value < 0.01; LSD). Scale bars = 10 mm

We found that seedlings that had been deprived of carbon for 4.5 days were able to regain their growth when transferred to light conditions only when sucrose was present in the medium (Fig. 5a, b). The recovery of seedling growth in these conditions was further illustrated by the development of LRs from the PR and of ARs from the hypocotyl, as well as by the substantial increase in their shoot area (Fig. 5b). The addition of sucrose to carbon starved seedlings was sufficient to trigger LR and AR recovery (Fig. 5c). Local application of 1% sucrose to the etiolated hypocotyl of carbon starved seedlings was sufficient for seedling growth recovery (Fig. 5d). To determine the specific requirement of sugars in the growth recovery observed in carbon starved seedlings, we transferred them to a medium supplemented with glucose, fructose, or sucrose (Suppl. Fig. S1). PR growth was successfully recovered on glucose, fructose, or sucrose medium under light conditions, but to a different extent depending on the type of sugar used (Fig. 5e). Interestingly, transfer of seedlings to a sucrose-containing media in the dark was sufficient to recover PR growth, which was mainly based on increased cell elongation (Fig. 5e). These results suggest that exogenously added sugars could contribute as direct sources of the carbon moieties required for the synthesis of cellular components driving growth of dark-grown seedlings.

### Sucrose dynamically alters heterochromatin characteristics and cell cycle progression in the RAM of carbon starved seedlings

**Fig. 6:**
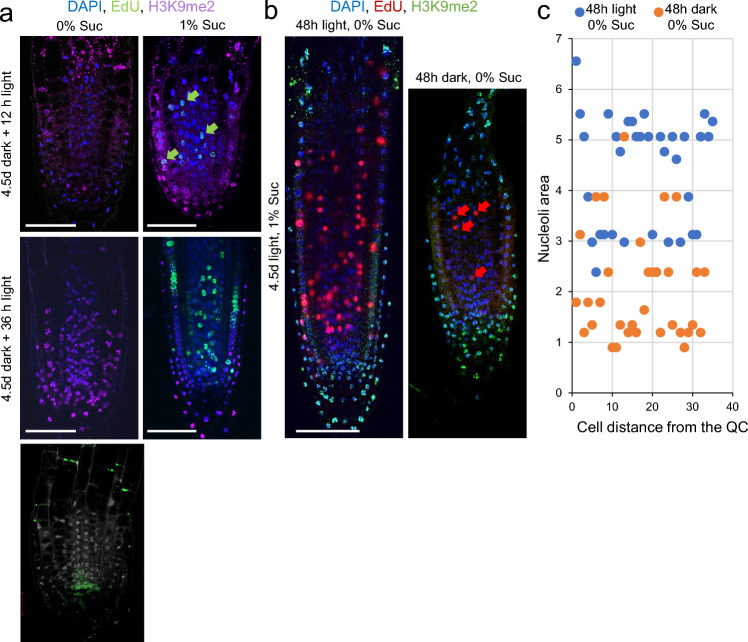
Sucrose dynamically alters heterochromatin features and cell cycle progression in the RAM of carbon-starved seedlings. **a** Changes in chromatin and cell cycle progression in response to sucrose were detected by EdU incorporation (green, arrows), DAPI staining (blue) and H3K9me2 immunolocalization (magenta). Seedlings in **a** have been grown in the dark for 4.5 days in the absence of sucrose and thereafter were transferred to liquid medium in light conditions with or without sucrose, as indicated. **b** Changes in chromatin and cell cycle progression were detected by EdU incorporation (red, arrows), DAPI staining (blue) and H3K9me2 immunolocalization (green). Seedlings have been grown in the light for 4.5 days with sucrose and thereafter were transferred to liquid medium for 48 h in dark or light conditions without sucrose. Seedlings in (**a**, **b**) were incubated in the presence of EdU and colchicine for 90 min prior fixation. **c** Nucleolus volume from seedlings in **b**. Scale bars = 40 μm

We next investigated the short-term effect of light and sucrose on chromatin characteristics within the RAM of 4.5-day-old carbon-starved seedlings (Suppl. Fig. S1). A short period (12–36 h) of incubation in light failed to induce DNA replication within the RAM in the absence of sucrose in the growth medium (Fig. 6a). It was found that transfer to light does not lead to recovery of root development. The addition of sucrose to the medium induced DNA replication of the cells within the RAM in the shortest incubation time of 12 h (Fig. 6a). Interestingly, H3K9me2 levels were reduced after 36 h of incubation in light only in the presence of sucrose in the growth medium (Fig. 6a). On the other hand, when seedlings that had been grown in light with 1% sucrose were transferred to the dark for 36–48 h without sucrose, a substantial reduction in the pool of DNA replicating cells (EdU positive cells) within the RAM was observed (Fig. 6b and Suppl. Fig. S6a, b). We found that H3K9me2 was restricted to the differentiated cells of the columella and lateral root cap and the EZ of the RAM either in dark- or in light-incubated seedlings without sucrose (Suppl. Fig. S4a, b) but appeared much closer to the QC in the case of the dark-incubated samples (Suppl. Fig. S6b). Furthermore, these cells contain significantly smaller nucleoli than those of light-incubated seedlings (Fig. 6c). Also, endogenous auxin levels were much lower in the RAM region of dark-incubated seedlings compared to light-incubated seedlings, as indicated by the higher expression of the DII-VENUS auxin sensor in the former (Suppl. Fig. S6a, b). Taken together, these results indicate that carbon starvation leads to reversible chromatin disruption of cell nuclei within the PZ of the RAM that reduces their proliferative potential and accelerates cell differentiation in the RAM. Our results also suggest that carbon starvation could have a direct effect on heterochromatin formation and endogenous auxin pool, which will indirectly drive cells in the PZ of the RAM to exit cell cycle and remain in G0.

### Effect of sucrose and auxin in seedling growth restoration after sugar starvation

**Fig. 7:**
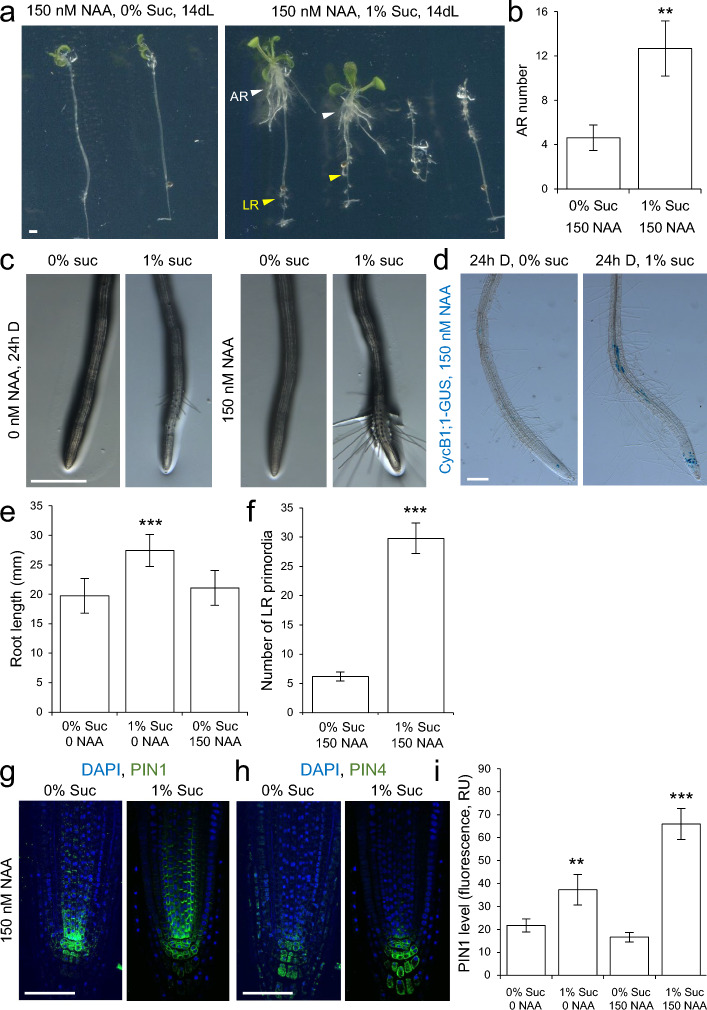
Effect of sucrose and auxin in seedling growth restoration after sugar starvation. **a** Seedlings were subjected to sugar starvation by growing them in the dark during 4.5 days without sucrose. Thereafter seedlings (**a–d**) or isolated root explants (**e–j**) were transferred to 0% or 1% sucrose medium supplemented with 150 nM NAA and grown in continuous light for several days. **b** Number of ARs after 10 days of growth in continuous light (10dL). **c**, **d** PR morphology of Col-0 (**c**) and of CycB1::GUS marker lines (**d**). Seedlings in **c** and **d** were incubated for 24 h in the dark on 0% or 1% sucrose medium with 150 nM NAA. **e**, **f** PR length (**e**) and number of LR primordia (**f**). **g**, **h** Localization of (**g**) PIN1 and (**h**) PIN4 in the RAM; green: PIN proteins and blue: DAPI. **i** Overall PIN1 levels in the RAM estimated from fluorescence. RU, relative units. PIN immunolocalization and quantification was done according to standard procedures (see Materials and methods). The bars in **b**, **e**, **f** and **i** show the mean ± SD values (*n* = 10). Asterisks indicate significant differences between treatments (*P*-value < 0.01; LSD). Scale bars = 2 mm (**a**, **c**, **d**) and 40 μm (**h**, **i**)

Application of exogenous auxin (150 nM 1-naphthaleneacetic acid, NAA) in the absence of sugar was not sufficient to restore PR growth and seedling development of carbon-starved seedlings after transfer to light conditions (Suppl. Fig. S7). On the other hand, 150 nM NAA induced AR formation in the etiolated hypocotyl region proximal to the shoot apex and LR formation in the PR either in the presence of glucose, fructose, or sucrose (Suppl. Fig. S7 and Fig. 7a, b). Interestingly, the known effect of exogenous auxin on premature cell differentiation in the EZ of the RAM (Moubayidin et al. 2010) was partially dependent on the presence of sucrose in the growth medium, as indicated by the development of root hairs near the tip of the root (Fig. 7c). In addition, cell cycle activation in the mature pericycle after NAA addition was also dependent in the presence of sucrose in the growth medium (Fig. 7d). Taken together, our results in carbon starved seedlings suggest that sugar mediates the local effects of exogenously applied auxin on PR growth and development.

To further assess the phenotype of exogenous application of NAA and its dependence on sugar addition to the medium, we incubated root explants isolated from 4.5-day-old seedlings, grown in light and with sucrose, with 0 and 150 nM NAA and 0% and 1% sucrose for 36 h (Suppl. Fig. S1). Consistent with our previous results, 150 nM NAA exerted PR growth inhibition and a significant increase in LR number only if sucrose was added to the medium (Fig. 7e–f). Similarly, PIN1, but not PIN4, protein levels in the RAM from root explants were reduced in the presence of 150 nM NAA only in the sucrose-free medium (Fig. 7g, h). We quantified PIN1 protein levels in the RAM of isolated root explants and found a significant increase in response to sugar and after incubation with 150 nM NAA (Fig. 7i). These results indicate a local effect of sugar in regulating PIN1 levels in the RAM.

## Discussion

Arabidopsis seedlings grown in the dark without sucrose showed reduced PR growth, which correlated with low numbers of dividing cells in the PZ of the RAM and changes in their nuclear and cellular morphology, suggesting premature differentiation of cells in the RAM. On the other hand, light and sucrose were found to maintain cell division in the RAM, which was sufficient to preserve RAM size and PR growth. Longitudinal zonation of the RAM has been extensively studied in many angiosperms and is known to depend on the tight balance between cell proliferation and cell growth (Ivanov and Dubrovsky 2013; Yamoune et al. 2021), which is based on a functional gradient of PLETHORA (PLT) proteins in the stem cell niche and the PZ of the RAM, as well as cytokinin-mediated downregulation of auxin signaling and transport in the EZ closest to the PZ, the so-called transition zone (TZ), through ARR12 (Di Mambro et al. 2017; Salvi et al. 2020). The PLT gradient is known to be the result of its auxin-induced expression around the QC region and its subsequent dilution in the PZ due to cell division (Mähönen et al. 2014). Auxin accumulation in the RAM is based on polar auxin transport from the shoot and on local auxin biosynthesis in the QC and stem cell region, both of which are strongly reduced in seedlings grown in the dark seedlings without sucrose (this work). We found that the stability of PIN1 and PIN2 in the RAM was mainly upregulated by sucrose and light, respectively. Therefore, dark-induced carbon starvation causes a plausible reduction in the ‘reverse-fountain’ auxin distribution pattern within the RAM (Grieneisen et al. 2007), which could explain the observed rapid cell differentiation in these conditions. In addition, the RAM of carbon-starved seedlings showed severe alteration of nuclei characteristics, such as reduction in nucleolus volume and reduced levels of the euchromatin marker H3K4me2, while the heterochromatin marker H3K9me2 revealed reprogramming of the meristematic nuclei in the PZ towards a heterochromatic state. Similar results were found in rice roots in response to abiotic stresses that led to a reduction in meristematic activity in the RAM (Polosoro et al. 2019).

Sugar-dependent PR growth was further demonstrated by transferring light-grown seedlings to dark conditions with and without sucrose, which caused a rapid (24 h) reduction in RAM length and a strong reduction in mitotic activity in the PZ. This was accompanied by a concomitant reduction in the size of the EZ and reduced growth during cell differentiation likely due to reduced endoreduplication levels. PIN1 and PIN7 stability. Thus, auxin levels in the RAM were strongly affected by sucrose and light. Our results suggest that sucrose is continuously required to maintain auxin homeostasis in the PR and for sustained cell proliferation and cell endoreduplication in the RAM. On the other hand, carbon-deprived seedlings transferred to light were able to recover PR growth and produce functional LR and ARs only in the presence of sugars in the growth medium. Carbon is the structural component of the synthesis of DNA and RNA molecules, hormones, and cellulose among other proteins. Therefore, carbon starvation can be considered as an upstream limiting factor for hormone metabolism, cell growth, cell cycle, and protein synthesis.

Previous work has shown that activation of the RAM and shoot apical meristem (SAM) during the heterotrophic-to-autotrophic transition is based on different sugar and light signals. The TARGET OF RAPAMYCIN (TOR) kinase acts as a central integrator of light and metabolic signals in response to photosynthesis-derived sugars (Ryabova et al. 2019). While glucose signaling is sufficient to activate TOR kinase in the RAM, both glucose and light are required for TOR activation in the SAM (Li et al. 2017). In response to photosynthesis-derived sugars, TOR phosphorylates E2 PROMOTER BINDING FACTOR (E2F) A in the RAM and promotes their activity in upregulating S-phase genes necessary for cell division (Xiong et al. 2013). TOR has recently been shown to be required to maintain root tip homeostasis in response to photosynthesis-derived sugars during the heterotrophic-to-autotrophic transition, likely through regulation of PLETHORA2 (PLT2) and PIN2 stability (Zhang et al. 2022). Furthermore, sugar-stimulated TOR activity in the root suppresses autophagy and maintains RAM activity to support root growth under nutrient-limiting conditions (Dong et al. 2022). Along with PR, sucrose also regulates LR development by inducing transcription of the *AUXIN RESPONSE FACTOR* and *LATERAL ORGAN BOUNDARIES DOMAIN* genes, downstream of the TOR pathway (Agrawal et al. 2023). These data position TOR as a gatekeeper for postembryonic PR growth and LR formation, through the integration of local auxin-dependent pathways with systemic metabolic (sugar) signals.

Our results clearly show that sucrose deprivation is related to precise cell cycle control in the RAM. The evolutionarily conserved SNF1-RELATED PROTEIN KINASE 1 (SnRK1) is a key regulator in the adjustment of cellular metabolism during starvation (Wurzinger et al. 2018), and is known to restrict growth through specific interactions with tissue-specific transcription factors (O’Brien et al. 2015). It is tempting to speculate that SnRK1 might also exert its negative role in cell cycle repression in the RAM during starvation through downregulation of PLETHORA (PLT) genes, a hypothesis that can now be tested. Alternatively, sucrose starvation might lead to overexpression of cell cycle inhibitors, such as the KIP-RELATED PROTEINS (Menges et al. 2005). Conversely, D-type cyclins play a key role in the transition from G1-to-S phase in response to hormonal and nutrient signals. In tobacco, *CycD3;4* expression was induced by sugar and its overexpression caused accelerated growth and a significant increase in the number of cells in the S and G2 phases (Kwon and Wang 2011). Interestingly, KIP-RELATED PROTEIN 2, which is known to specifically inhibit the kinase activity of the CycD2;1/CDKA;1 complex and to block the transition from the G1-to-S phase of the mitotic cycle (Sanz et al. 2011), is under the regulation of cytokinin in the transition zone (TZ) of the RAM to restrict the size of the meristem (Salvi et al. 2020).

Despite the well-known role of sugars in promoting auxin biosynthesis (Sairanen et al. 2013), our results indicate that light is the key factor required to induce local auxin biosynthesis in the QC and stem cell region, probably through upregulation of *YUC* expression. In addition, our results clearly indicate a positive effect of sugar on the stability of PIN proteins. Indeed, glucose-activated TOR phosphorylates and stabilizes PIN2 and thus affects the auxin gradient in the RAM (Yuan et al. 2020), suggesting a direct link between sugar sensing and the PIN-mediated establishment of the endogenous auxin gradient. On the other hand, we noticed that the known effect of exogenous auxin to induce pericycle division (and thus LR formation) and for premature cell differentiation in the EZ depended on the presence of sugar in the growth medium. These results suggest a complex regulation between auxin and sugars during root development (Mishra et al. 2022) that needs to be elucidated.

We found that local application of sugars to the etiolated hypocotyl of carbon starved seedlings was sufficient to restore PR growth, whereas the sugar-mediated effect on LR development in the PR and AR formation in the region of the hypocotyl near the shoot depended on the presence of light. Sugar-mediated growth recovery was primarily dependent on mitotic reactivation of cells within the RAM. However, growing seedlings longer than eight days in the dark without sucrose led to the irreversible differentiation of the cells in the RAM and the inability of sugar and light to recover seedling growth. In animals, the transition from proliferating cells to terminal differentiation requires a global downregulation of cell cycle genes via the formation of a transcriptional repressor complex called DREAM, which includes, among others dimerization partner, retinoblastoma and E2F proteins (Sadasivam and DeCaprio 2013). DREAM complexes can recruit chromatin modifiers to add repressive histone modifications at E2F-dependent cell cycle genes (Ma and Buttitta 2017). In the Drosophila wing during metamorphosis, changes in chromatin accessibility at essential and rate-limiting cell cycle genes provide additional barriers to mitogenic signals and provide a molecular explanation for the robust G0 state of postmitotic wing cells (Ma et al. 2019). Similar DREAM complexes that may be involved in the coordinated cell cycle and developmental regulation of E2F targets and mitotic genes have been identified in Arabidopsis (Magyar et al. 2016) and might play a similar role in the RAM during carbon starvation. Indeed, a DREAM complex has been recently described in Arabidopsis and inhibits H3K4me3 deposition on its target genes to repress their expression (Wang et al. 2022).

### Author contribution statement

TP designed and performed all the experiments. TP and SK analyzed the data. The first draft of the manuscript was written by TP, SK and KP. JMP-P and TP wrote the final manuscript. All authors read and approved the final manuscript.

### Supplementary Information

Below is the link to the electronic supplementary material.

Supplementary file1 (PDF 2593 KB)

## Data Availability

All data generated or analyzed during this study are provided in this published article and its supplementary data files or it will be provided upon a reasonable request.

## Abbreviations

AR: Adventitious root
E2F: E2 PROMOTER BINDING FACTOR
EZ: Elongation zone
LR: Lateral root
LSD: Least significant difference
NAA: 1-Naphthaleneacetic acid
PIN: PIN-FORMED
PR: Primary root
PZ: Proliferation zone
QC: Quiescent center
RAM: Root apical meristem
TOR: TARGET OF RAPAMYCIN
