# Supplementary material for: Regulation of early seedling establishment and root development in Arabidopsis thaliana by light and carbohydrates
Source: Planta. 2023 Sep 6;258(4):76. doi: 10.1007/s00425-023-04226-9 (PMC10480265; doi:10.1007/s00425-023-04226-9)
Supplement: Supplementary file 1 — Supplementary file1 (PDF 2593 KB) [file 425_2023_4226_MOESM1_ESM.pdf]

Supplementary Fig. S1

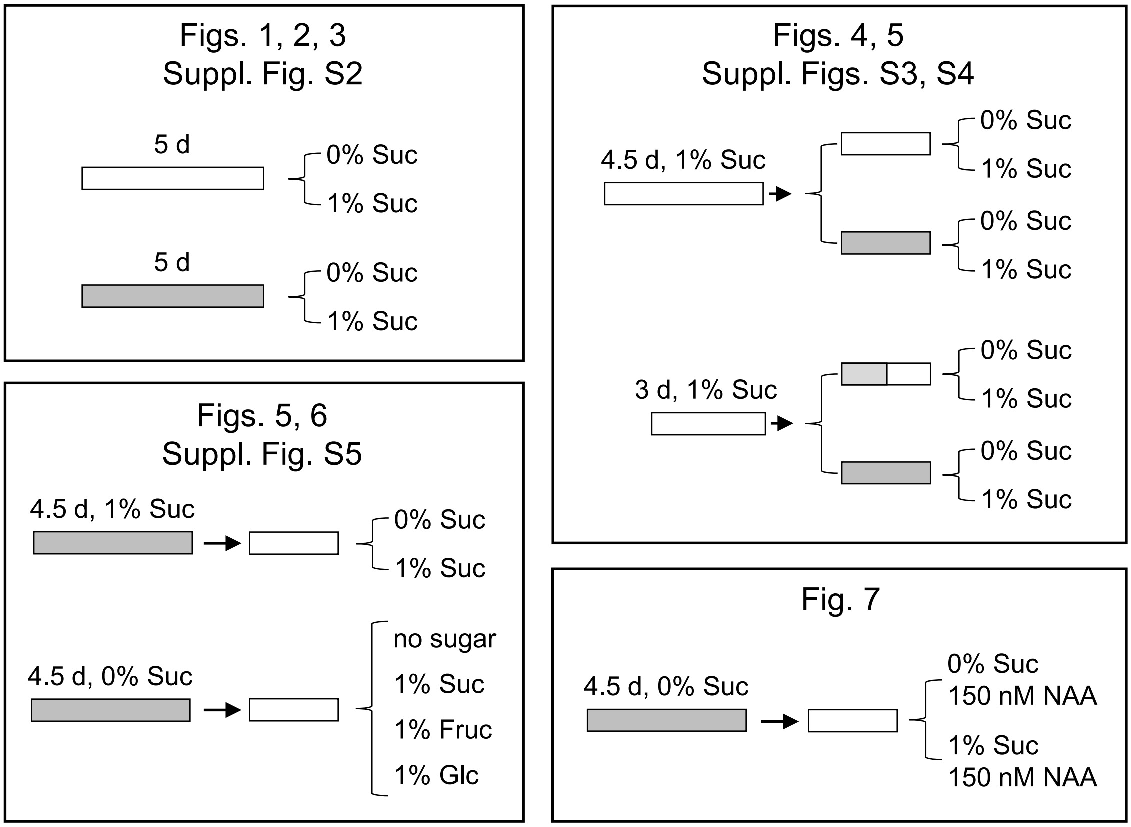

Supplementary Fig. S1. Experimental design used in this work. See the Materials and methods and Results sections for further details.

Supplementary Fig. S2

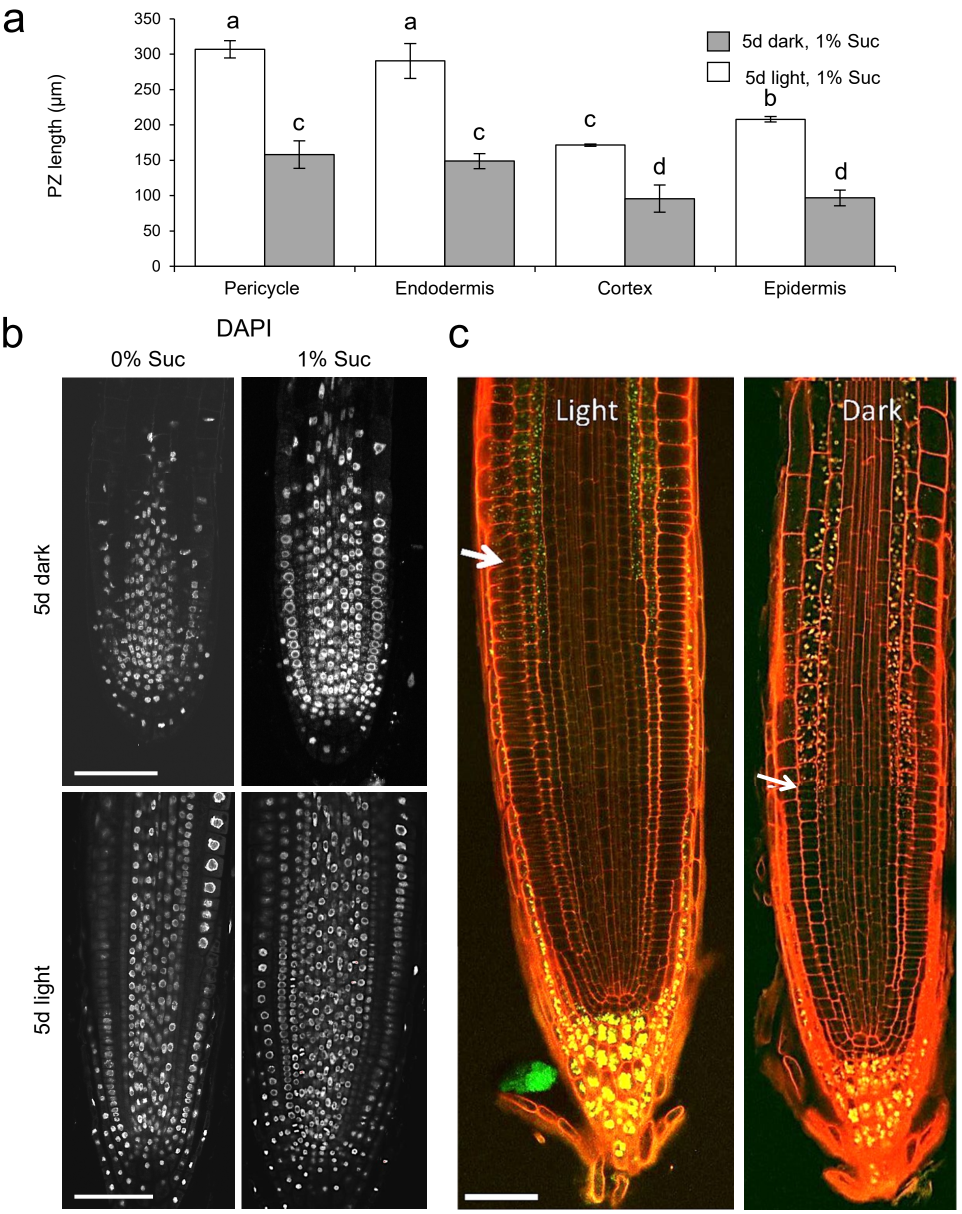

Supplementary Fig. S2. Quantitative analysis of the RAM structure under dark and light conditions with and without sucrose. Seedlings were grown 3 days under dark or light conditions, fixed, segmented and a cellular map of the outer cell layers was obtained as described elsewhere (Pasternak and Pérez-Pérez, 2021). **a** The relative positions of all mitoses were obtained for each cell type and the position of the most proximal mitosis within the RAM was considered to estimate the length of the PZ for each cell type. Letters indicate significant differences between treatments (*P*-value < 0.01; LSD). **b** Details of nuclei structure in the proximal RAM as stained by DAPI after 5 days in light or in dark conditions with and without 1% sucrose. **c** Polysaccharide labelling after 36 h incubation in the presence of sucrose. Starch granules are in yellow, while cell border is in red. Arrows shown starting point of the starch accumulation in the transition zone, which is correlated with cell elongation. Scale bars = 50 µm.

Supplementary Fig. S3

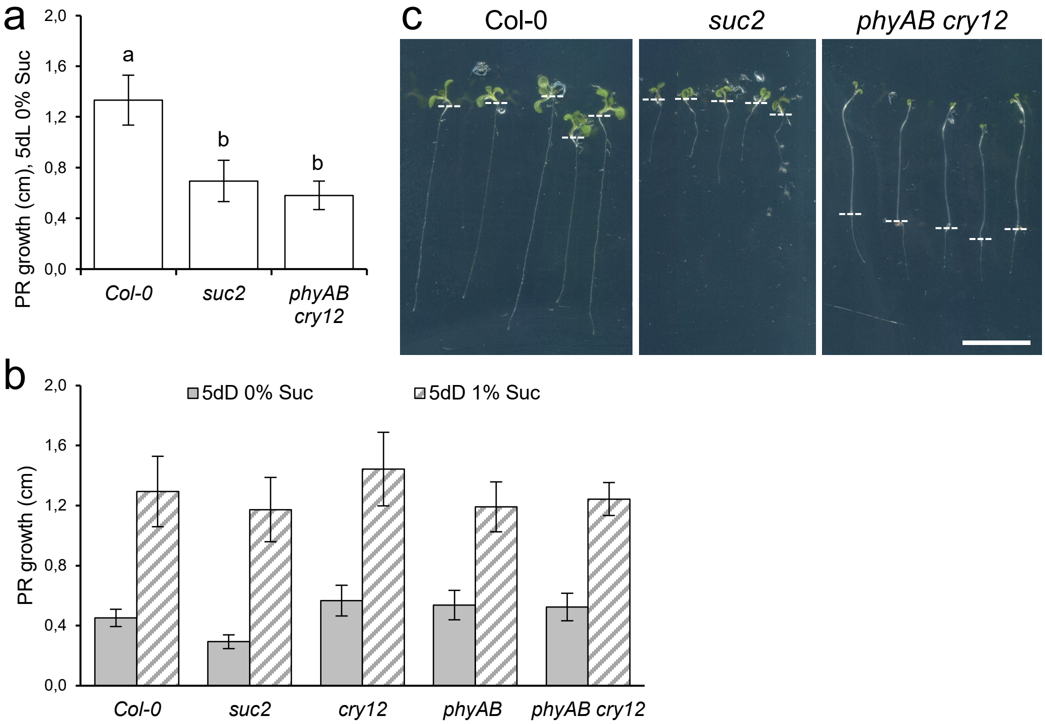

Supplementary Fig. S3. PR growth is dependent on photosynthesis-derived sugars. **a-b** Root length were measured in seedlings grown for 5 days in the medium with and without 1% sucrose, under 0 (Dark, D) or 100 µmol/m^2/sec continuous light (Light, L). Letters indicate significant differences between treatments (*P*-value < 0.01; LSD). **c** Representative images of seedlings grown under light conditions in medium without sucrose. Dashed lines indicate the hypocotyl-root junction in each seedling. Scale bar = 10 mm.

Supplementary Fig. S4

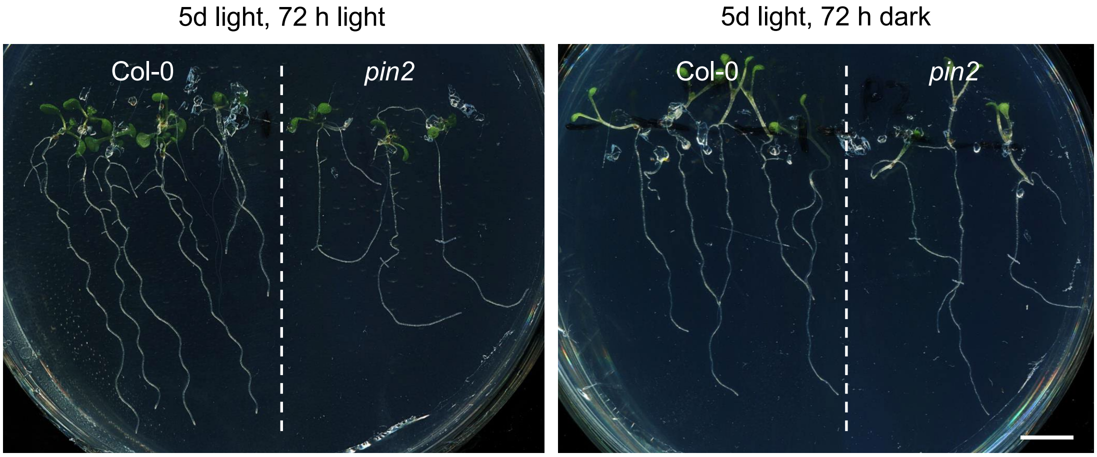

Supplementary Fig. S4. *pin2* mutant and WT seedlings were growth on the light with sucrose and thereafter *pin2* mutants were strength along the gravity vector and cultured in the dark or light for the next 48-96 h. Scale bars = 10 mm.

Supplementary Fig. S5

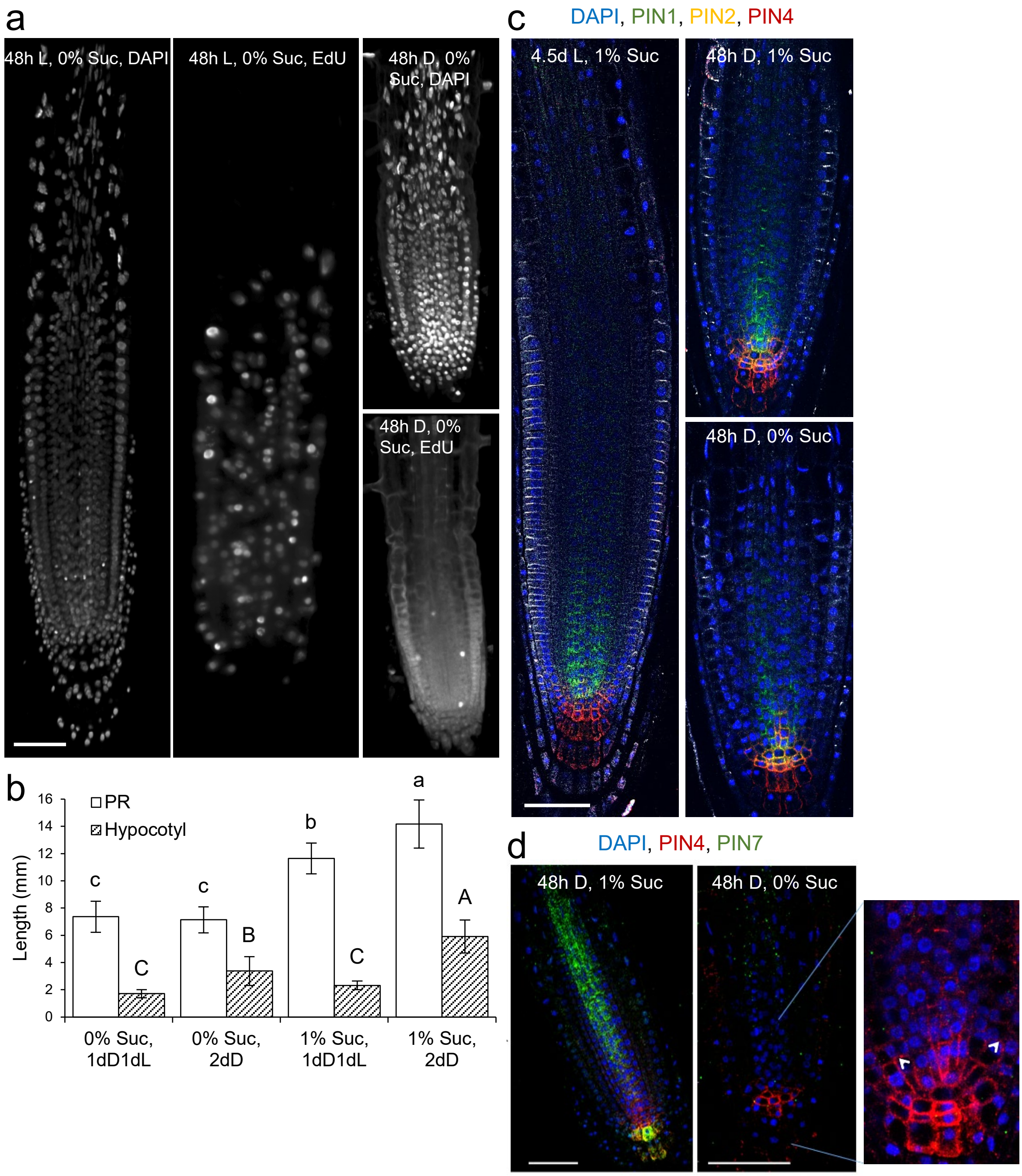

Supplementary Fig. S5. Post-germinative carbon starvation affects PR growth (cont.). **a** RAM structure of 4.5 days light-grown seedlings after 48 h of dark-incubation with and without sucrose stained with DAPI and EdU. **b** PR and hypocotyl length in seedlings grown for 3 days under LD conditions (16/8 h day/night) with 1% sucrose that were incubated in media with or without sucrose for 2 d in the dark (2dD) or for 1 d in the dark and 1 d in continuous light (1dD1dL). **c** Abundance of PIN1 (green, **c**), PIN2 (yellow), PIN4 (red) and PIN7 (green, **d**) proteins in the RAM of 4.5 days light-grown seedlings after 48 h of dark-incubation with and without sucrose. DAPI staining in shown in blue. The bars in **b** show the mean values ± SD. Letters indicate significant differences between treatments (*P*-value < 0.01; LSD). Scale bars = 40 µm.

Supplementary Fig. S6

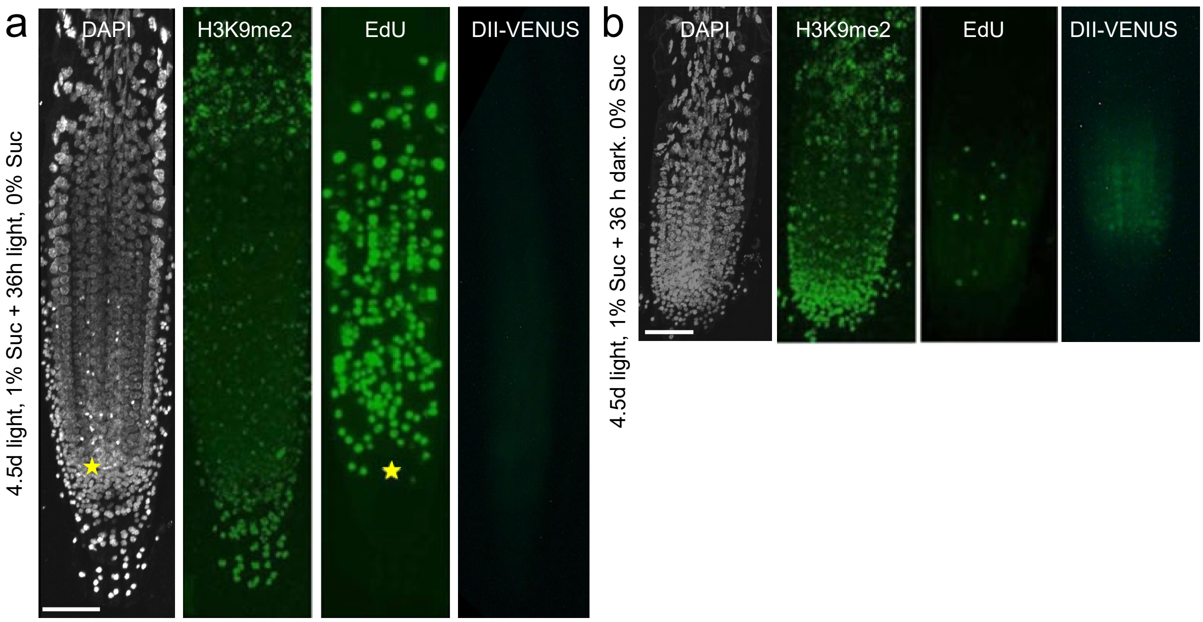

Supplementary Fig. S6. Sucrose dynamically alters heterochromatin features and cell cycle progression in the RAM of carbon-starved seedlings (cont.). Seedlings were grown in light in the presence of sucrose and then transferred to liquid medium without sucrose with further incubation in the dark or light for the next 36 hours. 10 µM EdU was added for 90 min, seedlings were fixed, EdU was detected and H3K9me2 localized. **a** Continuous light, without sucrose; **b** Dark without sucrose. Scale bars =: 40 µm.

Supplementary Fig. S7

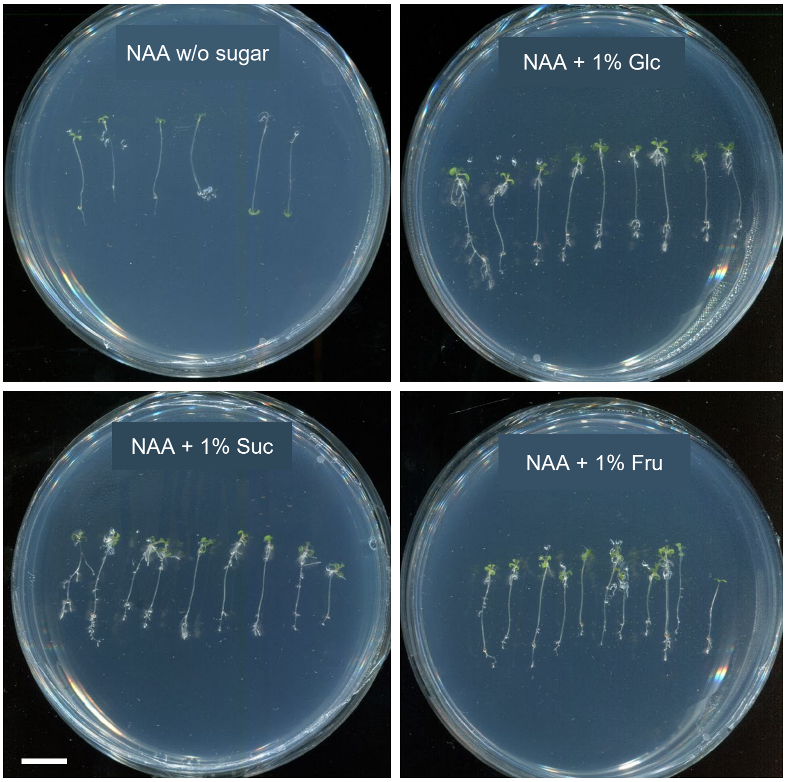

Supplementary Fig. S7. Effect of sugar and auxin in seedling growth restoration after sugar starvation. Seedlings were subjected to sugar starvation by growing them in the dark during 4.5 days without sucrose. Thereafter seedlings were transferred to plates containing 150 nM NAA and the indicated sugar supply. Images of whole seedlings were obtained after 6 days of growth in continuous light (10dL). Scale bar = 10 mm.
